# Editorial Correspondence

**Published:** 1878-02

**Authors:** R. W. Westmoreland

**Affiliations:** Washington, Ark.


### EDITORIAL CORRESPONDENCE.

Washington, Ark., Feb. 1, 1878.

*Editor Atlanta Medical and Surgical Journal:*

In your December issue, I see a letter from your European correspondent, which, on account of its practical importance, impressed me favorably. The subject, antisepticism, is one to which great attention has been devoted of late, resulting in decidedly valuable discoveries, it seems.

The economical value of the discovery that cotton, by filtering the atmosphere, prevents ingress of the septic material which causes putrescence in a vessel of milk, is certainly very great.

With the view of verifying the statement of Dr. Nunn by personal experiment, I prepared, according to his directions, a couple of bottles. Into these I put fresh sweet milk, and placing them in a water-bath, applied heat to the extent of 212° Farenheit—cotton having been placed over the mouths prior to heating, and every precaution taken against failure in the experiment. One bottle of the milk has been carefully preserved (the other having been broken in the experiment) for three weeks, and although the weather has been ususually warm for the season, I find upon opening the bottle and testing its contents, that the milk is apparently as fresh and sweet as when bottled. The only difference discoverable is consistency, it being thicker.

A very healthy season with us, and an acquaintance of only a few months in this locality affords me not a very large number of cases out of which to select for the theme of my letter. As "stricture" is a special subject to which several Atlanta surgeons have devoted much attention, I would like to hear from my friend, Dr. Shirley B., or some others, on the following case (though not exactly in their line) which, owing to my rather limited experience with obstetrical complications, gave me more annoyance than some "*sure enough*" cases I met with in Atlanta.

The subject, a white woman and multipara, was taken in labor, and partly on account of too early rupture of the membranes, and partly from large size of the child, the case proved vexatiously *tedious*. In addition to these hindrances, the pains were rather weak, but three grains of ergotine increased the throes in such disproportion to the relaxation of the os, that I gave a full dose of morphine. The labor terminated favorably in all things except obstinate retention of urine. No stricture ever closed the urethra more completely against the passage of the urine than this did without the catheter; and being deprived of the ocular advantages we have with the male, catheterism of this kind, to one not of long experience, is more laborious than using the urethrotome or divulcior.

The condition of the neck of the bladder and urethra causing retention in this case, is perhaps not very unusual when the head presses firmly for a long time against the pubis. Catheterism, sweet spirit of nitre and opium occasionally, however, I suppose, will be sufficient treatment till the parts recover from the effects of this mechanical injury. Respectfully, R. W. WESTMORELAND, M.D.

